# The link between breastfeeding profile and contraceptive use and barriers to breastfeeding: messages from a breastfeeding support center on the need for further strategies to improve breastfeeding

**DOI:** 10.3389/fped.2025.1633498

**Published:** 2025-11-06

**Authors:** Şenay Koçakoğlu

**Affiliations:** Faculty of Medicine, Department of Family Medicine, Harran University, Şanlıurfa, Türkiye

**Keywords:** breastfeeding, exclusive, contraceptives, family planning, maternal educational level, repeat cesarean birth, seasonal, work

## Abstract

**Background and objectives:**

The aim of this study is to evaluate the barriers to breastfeeding in a region with high fertility rates from the perspective of a breastfeeding support center (BSC). The broader aim of the study is to inform policymakers about the need to expand breastfeeding support centers and integrate family planning services (FPCs) into these centers.

**Methods:**

Data from 2,714 postpartum mothers from the tertiary hospital affiliated BSC in Şanlıurfa, Türkiye (2020–2024), were analyzed. Group comparisons were made based on contraceptive use, delivery mode, maternal educational level, and seasonal work status.

**Results:**

Contraceptive users had significantly better breastfeeding outcomes: The average duration of breastfeeding for previous first-born infants was significantly longer among contraceptive users (16.68 months, 95% CI: 15.10–18.26) compared to non-users (11.16 months, 95% CI: 10.02–12.30), *p* < .001.The mean duration of breastfeeding for all surviving children was also longer among contraceptive users (18.82 months, 95% CI: 17.20–20.44) vs. non-users (13.05 months, 95% CI: 11.78 to 14.32), *p* < .001.

**Conclusions:**

The findings of this study underscore the need to support breastfeeding, increase women's knowledge and awareness of contraceptive methods, and to enhance access to integrated family planning and breastfeeding counseling services, including via mobile and outreach modalities. Integrating FPCs into breastfeeding support centers (BSCs), particularly in regions with high fertility rates, low educational attainment, and where sociocultural norms strongly influence attitudes, can be a rational and effective strategy to improve maternal and child health by positively impacting breastfeeding outcomes.

## 1 Introduction

Improving breastfeeding practices is vital for enhancing public health. Breastfeeding significantly reduces infant mortality, malnutrition-related deaths, and long-term healthcare costs, while supporting motor, cognitive, and physical development (1, 2). WHO recommends exclusive breastfeeding (EBF) for the first six months and continued breastfeeding up to two years or beyond (3); however, breastfeeding rates persistently fall short of target levels (4, 5).

Systematic reviews of breastfeeding studies from diverse regions around the world have revealed that the initiation and maintenance of optimal breastfeeding are influenced by a wide array of factors. Factors such as economic and sociocultural constraints, lack of counseling, unsupportive workplace conditions, and insufficient support continue to act as significant barriers to breastfeeding (6–8). Despite heterogeneity among studies, the common conclusion is that breastfeeding requires strong support, and that strategies should be developed in accordance with regional contexts and the most prominent risk factors (9).

Addressing region-specific problems separately can be one of the solutions, and at this point, healthcare providers and communities must play a central role in promoting and sustaining breastfeeding efforts (10). It may also be rational to place greater emphasis on the use of contraceptive methods to prevent complications associated with frequent and multiple births, which can negatively affect breastfeeding as well as maternal and child health. Women of reproductive age may either be unaware of the principles of birth control or may opt not to use contraceptive methods for various reasons. According to the WHO, the contraceptive needs of millions of women aged 15–49 remain unmet globally (11).

It is essential to choose effective contraception methods during breastfeeding period. Progestin-only methods are preferred due to their minimal impact on lactation, while breastfeeding itself serves as a natural contraceptive via the LAM, with up to 98% efficacy under proper conditions (12–14).

Evidence supports that structured education provided to women during the antenatal period significantly enhances breastfeeding outcomes (15). Furthermore, education programs that included psychological components or were given in combination with postpartum support were found to be effective in extending breastfeeding beyond 12 weeks (16). Effective family planning (FP) counseling in the postpartum period also contributes to the protection of mother-baby health by prolonging the breastfeeding period as well as regulating fertility. According to Blazer and Prata's systematic review, women who are provided with contraceptive information and access services after childbirth significantly extend the time between births and reduce unwanted pregnancy rates (16, 17).

In Türkiye, almost all children are breastfed at some point, only 34% continue up to age two, and early initiation rates vary between rural (67%) and urban (73%) areas (18). With great efforts, breastfeeding counseling is routinely provided in family health centers starting from the eighth month of pregnancy and continues within maternity and pediatric clinics in hospitals where nurses observe and assist with breastfeeding immediately after birth in Türkiye. However, it is clear that due to time constraints and limited staffing, it is often difficult to fully integrate this service into daily clinical practice and the field should be supported by interventions (19).

Depending on literature more positive outcomes could be achieved if breastfeeding support were given greater priority and more dedicated time (20). Focused services can provide individualized support, regular educational updates, and sustained maternal engagement through reminder messages and follow-up calls, thereby encouraging continued breastfeeding and effective contraceptive use (21, 22).

This study addresses a critical gap in the literature by analyzing comprehensive data from Şanlıurfa-Türkiye's province with the highest fertility and migration rates (23) While the total fertility rate (TFR) in Türkiye has declined to 1.4 children per woman, well below the replacement level, the TFR in Şanlıurfa remains significantly higher at 3.28 (23). The influx of refugees due to humanitarian crises in surrounding regions stands out as one of the factors contributing to the rapid population growth in the region (24).

In some previous studies EBF rates in the first 6 months were reported to be between 15% and 26.5% (25–27) in Şanlıurfa. Although research in this region has provided valuable data on breastfeeding and contraceptive use profiles, as well as mothers' knowledge and attitudes, these topics have generally been studied separately or with fewer participants (26–28).

In this study, multiple prominent variables influencing breastfeeding outcomes in the region, with particular emphasis on contraceptive method use, are evaluated simultaneously using long-term, large-scale data from the perspective of a Breastfeeding Support Center (BSC) in Şanlıurfa. By highlighting the importance of synchronizing breastfeeding promotion with FPCs this study contributes a distinct and comprehensive approach that addresses two closely linked components of maternal and child health.

This study aims to: 1) To analyze the breastfeeding profiles of mothers who received health service from the BSC in Şanlıurfa; 2) To investigate how prominent factors in the region, especially the use of contraceptive methods, affect breastfeeding; 3) To provide evidence of the need to develop more effective strategies to protect and improve breastfeeding. The broader aim of this study is to inform policymakers in regions like this study area, where education levels are low and sociocultural norms play a role in shaping attitudes, about the need to increase the number of BSCs served by full-time, focused, certified staff and to integrate FPCs into these centers. The findings of this study may help elevate breastfeeding rates to optimal levels and prevent the adverse health consequences of frequent and high-parity births for mothers and infants.

## 2 Materials and methods

### 2.1 Study design

This retrospective, analytic and cross-sectional study was approved by the Harran University Clinical Research Ethics Committee (26.08.2024 date, HRU 24.12.12). All data collected in this study were kept strictly confidential and used solely for research purposes. Participants’ identities were anonymized and were not disclosed at any stage of the research. The study was conducted in accordance with the principles of the Declaration of Helsinki and relevant national ethical guidelines.

The study was conducted in the BSC, affiliated with the Department of Family Medicine at Harran University Hospital. Since 2018, this center has been providing structured breastfeeding education and support services to mothers. The breastfeeding profile of mothers in a high TFR region as well as the effect of CS/Ds, educational level and seasonal work status on breastfeeding outcomes, are focused.

### 2.2 Data collection tool

Study data were obtained from data collection forms (YDR.33) used for routine follow-up and training purposes which contains questions about the mothers’ obstetrical, breastfeeding, and contraceptive use status. It is not as a standardized or psychometrically validated scale and no scores could be assigned to the answers of the participants (see Supplementary Material S1).

### 2.3 Definitions

#### 2.3.1 Contraceptive methods

Contraception provide individuals the chance to decide for their sexual and reproductive health issues with high knowledge and awareness. Contraceptive methods are widely classified as traditional (e.g., coitus interruptus, calendar method, LAM and modern (combined oral contraceptives, minipills, male condom, intrauterine device) methods (30). In this study, LAM was separated in tables and in-text representations according to the study design due to its more effectiveness and wider preference among other methods in the region.

#### 2.3.2 Exclusive breastfeeding (EBF)

The term EBF “refers to the infant's receiving only breast milk, no other liquids or solids (or even water) other than oral rehydration solution or vitamin, mineral, or drug drops/syrups” (3).

#### 2.3.3 Breastfeeding barriers

Breastfeeding barriers refer to the factors that adversely affect the initiation or continuation of breastfeeding **(**e.g., sociocultural, financial, mental and physical issues). This study focused on sociodemographics as well as lack of contraceptive method use, CS/Ds, seasonal work factors (9).

### 2.4 Study population and sample selection

The study population included mothers who received breastfeeding support services at the accredited BSC between 2020 and 2024 in Şanlıurfa. A total of 6,264 applications were initially recorded. Although the sample size had been calculated *a priori* to ensure efficiency, it was decided to evaluate the entire dataset of mothers who met the inclusion criteria so as to maximize statistical power and generalizability.

**Inclusion criteria** were as follows:

- Mothers over 18 years old without certain medical conditions that affect breastfeeding ability;
- Mothers who have previous breastfeeding experience;
- Full-term mothers (37-42 weeks).

**Exclusion criteria** were as follows:

- Mothers under 18 years of age;
- Preterm delivery;
- Certain medical conditions affecting breastfeeding;
- First-time mothers and mothers who had not any breastfeeding experience)

Many pregnancies and births in Şanlıurfa are high risk or complicated, and thus referred to hospitals. As BSC is affiliated with a tertiary care hospital, mothers applying here are generally and disproportionately those who delivered by cesarean section under medical indication, including repeated cesarean sections. According to Şanlıurfa Provincial Health Directorate birth statistics (29), in hospital cesarean-delivery rate in Şanlıurfa is quite high which helps explain why in our sample the cesarean rate approaches ∼97%. Only a small fraction of deliveries are occurring in low-risk settings or primary care, which are less likely to be referred to our center.

### 2.5 Sample size and power analysis

*A priori* power analysis was conducted using G*Power 3.1 software. The following parameters were used:

**Table:** 

Parameter	Value
Effect size (Cohen's *f*)	0.25 (medium)
Significance level (α)	0.05
Desired power (1−β)	80% → required sample size ≈ **270**
Desired power (for higher sensitivity)	95% → required sample size ≈ **400**

Since data from all mothers who met the inclusion criteria were analysed, the final sample size was *n* = 2,714, which far exceeds the minimum required size and thus provides adequate power for all planned analyses.

### 2.6 Statistical analysis

Statistical analyses were performed using SPSS version 23.0 (IBM Corp., Armonk, NY, USA). The distribution of quantitative variables was assessed through evaluations of skewness and kurtosis coefficients, as well as the Kolmogorov–Smirnov test. Variables demonstrating acceptable conformity with the normal distribution were reported as mean ± standard deviation. For group comparisons involving normally distributed continuous variables, independent samples t-tests or One-Way ANOVA were used as appropriate. However, when the normality assumption was not met or when sample sizes were insufficient for parametric testing, non-parametric alternatives were employed. Specifically, the Mann–Whitney *U* test was used to compare two independent groups, as it does not assume normal distribution and is more suitable for ordinal or non-normally distributed data. For comparisons across three or more groups, the Kruskal–Wallis test was applied in similar non-parametric cases.

Categorical variables were presented as frequencies (*n*) and percentages (%). To examine associations between categorical variables, chi-square tests were primarily used. However, when expected counts in one or more cells of the contingency tables fell below 5—violating chi-square test assumptions—the Monte Carlo simulation method was applied to obtain more reliable p-values. This simulation was conducted with 10,000 iterations at the 95% confidence level. Effect sizes for significant associations were reported using eta squared (*η*^2^) where applicable.

The distribution of missing data was examined and ranged from 0.4% to 4.1%. To determine whether the missingness was random, Separate Variance t-tests, Little's MCAR test, and tabulated missing data patterns were analyzed. Based on these analyses, the data were deemed missing completely at random (MCAR). As a result, missing values were imputed using the mean for normally distributed variables and the median for non-normally distributed variables, ensuring the integrity of the dataset for subsequent analysis. A significance level of *p* < 0.05 was considered statistically significant for all tests.

## 3 Results

**Figure 1:**
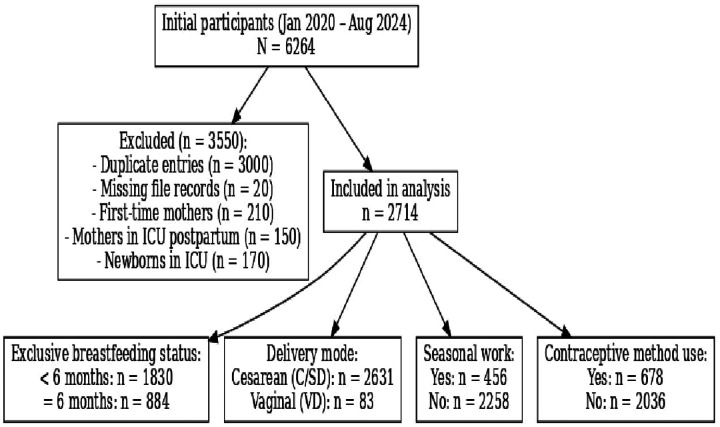
Flowchart of inclusion and exclusion process and brief outcomes.

Among the 6,264 mothers screened, 3,550 were excluded based on predefined eligibility criteria, resulting in a final analytical sample of 2,714 mothers, as summarized in Figure 1.

### 3.1 Relationship between sociodemographic/clinical factors and contraceptive use

Contraceptive use was found to be significantly associated with variables such as occupation (*χ*^2^ = 36.462, *p* < 0.001) education level (*χ*^2^ = 217.996, *p* < 0.001), duration of breastfeeding for the first child (*χ*^2^ = 344.564, *p* < 0.001), and the most recent mode of delivery (*p* = 0.003). In particular, women who exclusively breastfed for only the first 6 months were observed to have higher rates of contraceptive use. However, no significant association was found between the timing of breastfeeding initiation and postpartum contraceptive use (*p* = 0.840) (Table 1). These findings suggest that contraceptive use is influenced by personal, sociocultural and breastfeeding-related factors.

**Table 1:** Relationship between sociodemographic/clinical factors and contraceptive use.

Variable	No contraceptive use, *n* (%)	Contraceptive use, *n* (%)	Chi-square Monte Carlo, (%99 CI lower-upper)	*p*-value^a^
Occupation			36.462, (<0.001–<.001)	<0.001
Housewife	1,643 (80.7)	615 (90.7)		
Seasonal worker	393 (19.3)	63 (9.3)		
Education level			217.996 (<0.001–<.001)	<0.001
Illiterate	993 (48.8)	203 (29.9)		
Primary school	879 (43.2)	301 (44.4)		
Middle school	46 (2.3)	16 (2.4)		
High school	84 (4.1)	72 (10.6)		
University or higher	34 (1.7)	86 (12.7)		
Time of first breastfeeding			–	0.840
Within the first hour	733 (36.0)	247 (36.4)		
After the first hour	1,303 (64.0)	431 (63.6)		
First child's breastfeeding duration			344.564 (<0.001–<.001)	<0.001
< 6 months(any)	475 (23.3)	92 (13.6)		
6 months(any)	256 (12.6)	16 (2.4)		
7–11 months	292 (14.3)	54 (8.0)		
12 months	351 (17.2)	42 (6.2)		
13–17 months	113 (5.6)	49 (7.2)		
18 months	243 (11.9)	130 (19.2)		
19–23 months	65 (3.2)	69 (10.2)		
24 months	226 (11.1)	208 (30.7)		
≥ 25 months	15 (0.7)	18 (2.7)		
Most recent delivery mode			–	0.003
Cesarean section	1,962 (96.4)	669 (98.7)		
Vaginal delivery	74 (3.6)	9 (1.3)		
Exclusive breastfeeding for first baby			–	<0.001
<6 months EBF	1,596 (78.4)	234 (34.5)		
=6 months EBF	440 (21.6)	444 (65.5)		

a Chi-square test was used for all comparisons.

### 3.2 Relationship between contraceptive use and breastfeeding durations, sociodemographic factors, delivery mode

Comparative analysis of contraceptive use and breastfeeding durations in Table 2 show contraceptive users were slightly older on average (32.6 vs. 31.2 years; *p* < 0.001, Cohen's d = 0.26), and had fewer miscarriages, vaginal deliveries, and cesarean sections, although these differences had small effect sizes (Cohen's d = 0.14–0.29). In contrast, breastfeeding durations showed much stronger associations. Women who used contraception had notably longer breastfeeding durations for their first child (16.7 vs. 11.2 months; *p* < 0.001, Cohen's d = 0.74), and higher average breastfeeding duration for all living children (18.8 vs. 13.1 months; *p* < 0.001, Cohen's d = 0.90). Results displayed in Table 2 suggest that while demographic and obstetric variables had a limited association, breastfeeding practices were more strongly linked to postpartum contraceptive use.

**Table 2:** Comparison of sociodemographic factors, delivery mode, and breastfeeding durations by contraceptive use.

Variable	Contraceptive use		Cohen's D effect size	*p*-value^a^
	No mean ± SD/median (Min–Max)	Yes mean ± SD/median (Min–Max)		
Age (years)	31.16 ± 5.77/31 (18–56)	32.62 ± 4.69/33 (20–51)	−0.26	<0.001
Number of miscarriages	0.65 ± 1.21/0 (0–11)	0.43 ± 0.84/0 (0–5)	0.19	<0.001
Number of vaginal deliveries	1.16 ± 1.91/0 (0–13)	0.63 ± 1.28/0 (0–9)	0.29	<0.001
Number of cesarean deliveries	3.80 ± 1.83/4 (0–9)	3.54 ± 1.52/4 (0–8)	0.14	<0.001
First child's breastfeeding duration (months)	11.16 ± 7.46/11 (0–72)	16.68 ± 7.81/18 (1–36)	−0.74	<0.001
Average breastfeeding duration of living children (months)	13.05 ± 6.50/12 (0–30)	18.82 ± 5.61/18 (1–36)	−0.90	<0.001

a Mann–Whitney *U* test was used for all comparisons.

### 3.3 Relationship between EBF and education level

There was a statistically significant association between maternal education level and EBF duration (*χ*^2^ = 61.884, 99% CI: <0.001 to <0.001, *p* < 0.001). Mothers with EBF duration of less than 6 months were more frequently illiterate (47.7%) or had only primary education (42.8%) (Table 3). This finding suggests that higher maternal education is positively correlated with prolonged EBF, possibly reflecting increased awareness of breastfeeding benefits and better access to health information among more educated mothers.

**Table 3:** Comparison of seasonal work, education level, and contraceptive use by exclusive breastfeeding.

Variable	Exclusive BF <6months *n* (%)	Exclusive BF = 6 months *n* (%)	Chi-square Monte Carlo, (%99 CI lower-upper)	*p*-value^a^
Seasonal agriculture wok			–	<0.001
No	1,392 (76.1)	866 (98.0)		
Yes	438 (23.9)	18 (2.0)		
Education level			61.884 (<0.001–<.001)	<0.001
Illiterate	872 (47.7)	324 (36.7)		
Primary school	784 (42.8)	396 (44.8)		
Middle school	38 (2.1)	24 (2.7)		
High school	83 (4.5)	73 (8.3)		
University or higher	53 (2.9)	67 (7.6)		
Contraceptive method use			–	^b^
None	1,596 (87.2)	440 (49.8)		
Traditional methods (other than LAM)	12 (0.7)	12 (1.4)		
LAM	186 (10.2)	388 (43.9)		
Condom	34 (1.9)	37 (4.2)		
Pill	2 (0.1)	6 (0.7)		
Intra Uterin Device	0 (0.0)	1 (0.1)		

a Chi-square test was used for all categorical comparisons.

b *p*-value not calculated due to violation of Chi-square assumptions. The expected frequencies was not greater than 5.

### 3.4 Relationship between contraceptive use by EBF

The analysis revealed a significant association between contraceptive method use and EBF duration. Mothers who exclusively breastfed for less than 6 months predominantly reported not using any contraceptive method (87.2%), whereas nearly half (49.8%) of those who exclusively breastfed for 6 months used no contraception. Notably, 43.9% of the latter group preferred LAM. Usage rates of other contraceptive methods, including condoms, pills, and intrauterine devices, were minimal across both groups (Table 3).

Although the chi-square test could not be calculated (Monte Carlo *p*-value unavailable), the marked differences in contraceptive choices suggest a strong relationship with breastfeeding duration. The pattern indicates that extended EBF is associated with a higher preference for natural contraception methods such as LAM, potentially due to concerns over hormonal or device-related interventions during the breastfeeding period.

### 3.5 Relationship between seasonal work and EBF

**Figure 2:**
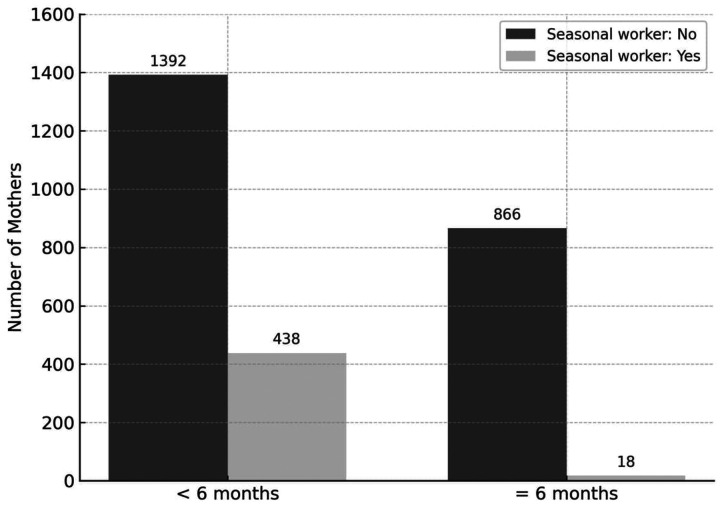
Distribution of exclusive breastfeeding duration for six month by seasonal work Status.

A statistically significant association was observed between seasonal agricultural work and EBF duration (*p* < 0.001). Mothers engaged in seasonal agricultural labor were considerably less likely to exclusively breastfeed for the recommended 6 months. The Monte Carlo simulation of the chi-square test confirmed this association, although confidence intervals could not be calculated due to sparse data (Table 3). The distribution of EBF by seasonal work can be seen in Figure 2. These findings suggest that participation in seasonal agricultural work may negatively impact the likelihood of maintaining EBF for the recommended 6-month period.

### 3.6 Distribution of breastfeeding duration for first-born infants

**Figure 3:**
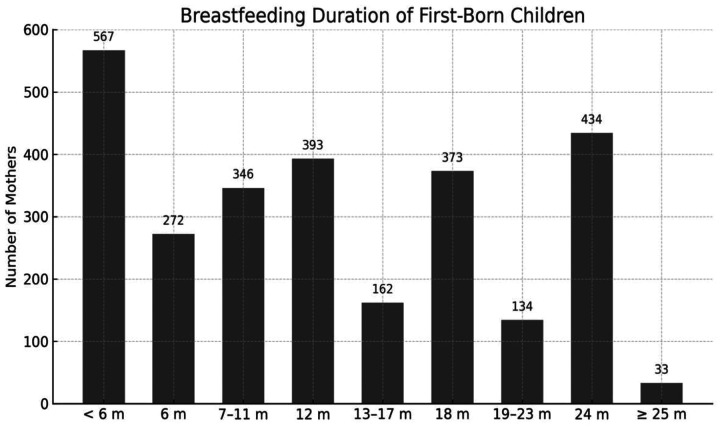
Distribution of breastfeeding duration for first-born infants (*n* = 2,714). M,months.

In the total sample of 2,714 mothers, 434 (15.99%) breastfed their first child for up to 24 months, and 33 (1.21%) for 25 months or longer (Figure 3).

First-time mothers may have more time, motivation, or social pressure to follow recommended breastfeeding practices, potentially explaining the relatively higher percentages of breastfeeding for 24 months or longer. They may receive more focused attention from healthcare providers or family members, which could support longer breastfeeding durations.

### 3.7 Distribution of mean breastfeeding duration of the first child and overall children by contraceptive Use

**Figure 4:**
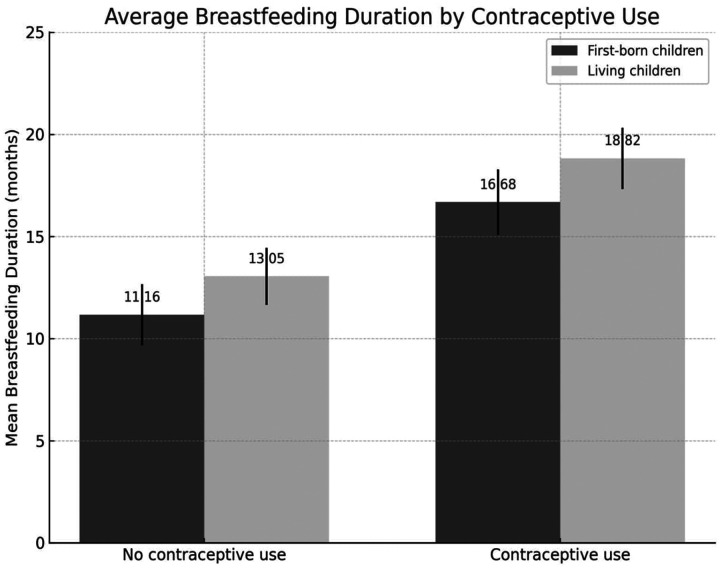
Distribution of mean breastfeeding duration of the first child and overall children by contraceptive Use.

Both mean breastfeeding duration of the first child and the overall mean breastfeeding duration were higher among mothers who used contraceptive methods (Figure 4).

### 3.8 Distribution of first breastfeeding time by delivery mode

**Figure 5:**
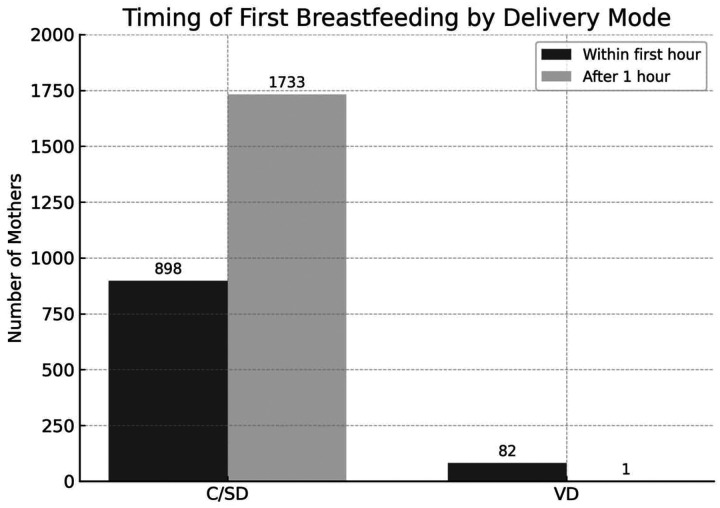
Timing of first breastfeeding by mode of delivery. VD, vaginal delivery; C/SD, cesarean section delivery.

The timing of breastfeeding initiation differed markedly by mode of delivery. Among those who delivered via CS/D (*n* = 2,631), 1,733 (65.9%) initiated breastfeeding after the first hour postpartum, while only 898 (34.1%) did so within the first hour. In contrast, nearly all mothers who had a vaginal delivery (*n* = 83) initiated breastfeeding within the first hour (*n* = 82; 98.8%), These findings suggest that cesarean delivery is associated with delayed initiation of breastfeeding, highlighting the need for targeted support and interventions to promote early breastfeeding among cesarean-delivered mothers (Figure 5).

## 4 Discussion

In line with international evidence (6–9, 31, 32) this study in Şanlıurfa underlines that limited maternal education, high rate of cesarean section, low use of contraceptive methods, and socioeconomic constraints such as seasonal work are key barriers to early initiation and EBF.

### 4.1 Maternal age

Maternal age was included as a key variable in our study because of its well-documented influence on breastfeeding practices, including initiation, duration, and exclusivity, through intertwined biological, psychological, and social mechanisms. Literature suggests that older mothers tend to breastfeed for longer durations, but much of this association is mediated by parity and educational attainment rather than age alone (33–35). In our findings, younger mothers used significantly lower levels of contraception and had more miscarriages compared to older mothers. In a setting such as Şanlıurfa, where modern contraceptive use and access to FPCs remain limited, this pattern has broader implications: older mothers are more likely to space births and avoid some obstetric complications, which promotes maternal recovery and allows for more sustained breastfeeding. Some studies reflect similar findings (33), while others show differing trends (34), often depending on the region's sociocultural norms. For example, in the United States, a study showed that the positive association between increased maternal age and longer breastfeeding duration was largely mediated by higher parity and education rather than age itself (35).

### 4.2 Maternal education level

Maternal education has been consistently implicated in global literature as a key determinant of breastfeeding initiation and duration. For instance, Tang et al. in China demonstrated that higher education increases the likelihood of early breastfeeding initiation, yet paradoxically is associated with lower EBF rates for the first six months, particularly among more educated working mothers (36). Similarly, a systematic review and meta-analysis reported that while education supports early initiation, its positive effects may be mitigated once factors like employment, socioeconomic status, and cultural norms intervene (37).

In this study, we found concordant trends: very low maternal education was strongly correlated shorter EBF duration. However, our setting exhibits a unique clustering of risk factors—high cesarean and miscarriage rates, low contraceptive use, seasonal work—that amplify the negative impact of low education more visibly than in many studies from United Arab Emirates and (UAE) China (32, 36). These observations suggest that in contexts with compounded obstetric and socioeconomic vulnerabilities, education alone may be necessary but not sufficient; it must be integrated with broader health, reproductive, and social interventions to substantially improve EBF outcomes. It is concluded that well-educated women can access information resources and services more easily. Moreover, they can be more advantageous in learning and developing attitudes, more motivated to use contraceptive methods by being aware of the harms of multiple births at frequent intervals on the health of the mother and baby. As a matter of fact, in line with the literature (36, 38), this study revealed that there is a strong relationship between high education level and contraceptive use, and this situation was interpreted with the advantages of being well-educated, as emphasized above. However, this may have a negative impact on the continuation of breastfeeding for ideal periods, because the early return of women with a high level of education to active working life constitutes a significant obstacle to the continuation of breastfeeding. This underscores the complex interplay between education, contraception, and breastfeeding behaviors.

### 4.3 Cesarean delivery

In presented study, high rates of cesarean deliveries were observed, which was associated with delayed initiation of breastfeeding or reduced continuation. This aligns with findings from O'Connell et al.'s meta-analysis in the UAE and Perrella et al.'s in Australia (7, 39) where cesarean delivery was identified as a negative factor for both early initiation and maintenance of EBF. In the cross-sectional study by Al Sabbah et al. in Dubai, mixed feeding was significantly more common among mothers who had cesarean births, indicating that cesarean delivery adversely affects EBF (32).

In our study, the unusually high cesarean rate can be attributed to several interrelated factors. Firstly, many pregnancies in Şanlıurfa are classified as high-risk, often due to complications or prenatal conditions, which increases the need for cesarean delivery to ensure maternal and fetal safety. Secondly, our study center (BSC) is affiliated with a tertiary care hospital, resulting in a referral bias: more complicated and higher risk cases are sent to this institution, including patients with prior cesareans or obstetric complications, which naturally elevates the proportion of cesarean births. Thirdly, medical indications such as fetal distress, cephalopelvic disproportion, placenta previa, or other obstetric pathologies frequently drive the decision toward cesarean delivery, especially when vaginal delivery is deemed risky. Taken together, these conditions reflect both the pregnancy profile of the population served and the healthcare referral structure in the region; thus, the high rate does not necessarily reflect overuse but rather the concentration of high-risk cases and medical necessity in a tertiary hospital setting. This effect is amplified by very low maternal education and other reproductive health and socioeconomic barriers, making the impact on breastfeeding outcomes more pronounced in our context.

CS/D showed a significant relationship with contraceptive use. We observe the same interventions and agree with the comment that women undergoing cesarean sections—who often have closer surgical follow-up and counseling—receive more structured postpartum contraceptive guidance (33). Encouragingly in line with literature (40), many cesarean mothers in our study eventually established breastfeeding, indicating that dedicated lactation support and early skin-to-skin contact even in surgical settings can mitigate this delay These findings support the need for cesarean section-specific breastfeeding protocols in hospitals to bridge the gap between mode of delivery and initiation of feeding (41). A similar trend was identified in a study from Uganda, where cesarean delivery was independently associated with a significant increase in immediate postpartum contraceptive uptake (42). These findings may be explained by the more intensive clinical engagement that typically accompanies cesarean births—longer hospital stays, increased surgical follow-up, and routine postpartum counseling—creating a strategic window for contraceptive counseling and method provision. Importantly, these contexts allow health providers to address the need for adequate birth spacing and postoperative recovery, framing FP as a critical component of maternal care.

The findings of this study, supported by the literature (43), revealed a clear, intrinsic link between contraception counseling and breastfeeding support. A key implication is the need to expand the number of BSCs and to integrate FPCs into these centers. Barriers to postpartum FP—such as misconceptions about contraceptive safety during lactation—can be more effectively addressed in centers that already provide breastfeeding support and peer counseling. Doing so would strengthen postpartum care, promote healthy birth spacing, and improve both maternal and infant health outcomes.

### 4.4 Breastfeeding for first child

According to the literature first-time mothers, widely due to lack previous exposure to breastfeeding, may feel uncertain or anxious about their ability to nurse effectively (44). Low breastfeeding self-efficacy has been consistently linked with early cessation of breastfeeding. Nipple pain or trauma, delayed onset of lactation can lead them to early supplementation with formula.

In this study, the duration of breastfeeding of mothers’ first babies was found to be below ideal levels and one of the reasons can be not having adequate information about breastfeeding. Supportingly Choedon et al.'s study revealed that primiparous mothers had generally insufficient knowledge about EBF (45). According to the findings of this study, approximately one in five mothers with their first breastfeeding experience continue to breastfeed their child for up to 24 months, which is particularly promising. Possible explanations for this outcome include that mothers in their first breastfeeding experience may possess stronger motivation, have more time available, and receive more focused support from healthcare providers or family members, all of which can contribute to longer breastfeeding durations.

### 4.5 Contraceptive use

In this study, contraceptive use, and preference for modern methods among mothers were found to be very low. Similar findings have been reported in literature; reasons often cited include fear of side effects, lack of information, the return of menstruation being used as a marker for when to adopt contraception, weak partner support, and limited access to preferred modern methods (46). The main reason behind their lack of protection in study area may be the belief that mothers with many children will have more power (47). However, the fact that multiple births at frequent intervals pose a major threat to the health of the mother and the baby should not be overlooked, and these points should be emphasized when providing counseling.

A key positive finding of this study is the strong association between postpartum contraceptive use, particularly the LAM, and improved breastfeeding outcomes. LAM not only facilitates birth spacing but also reinforces the practice of EBF through behavioral conditioning (48). Lactation is regulated hormonally by prolactin—essential for milk production in response to postpartum hormonal shifts and infant suckling—and oxytocin—which triggers milk ejection and supports emotional bonding and maternal relaxation. Recent evidence shows that efficient oxytocin release, especially pulsatile runs in early postpartum, correlates with higher milk output and longer breastfeeding duration, while maternal stress dampens this response (49). However, mothers should be made aware that starting supplementary foods reduces the effectiveness of this method. Increasing the awareness of mothers and mother candidates about not only the contraceptive aspect of breastfeeding but also all its unique benefits on mother and baby health can contribute positively to improving breastfeeding rates (50). LAM is reported to be, when correctly practiced, reliable and is often preferred due to its natural, hormone and intervention free approach (51). Our data supports global research indicating that women who use LAM tend to breastfeed more likely to achieve six months of EBF (52).

It is reported that effective breastfeeding as a contraceptive method can provide protection up to 98% compared to many traditional methods if amenorrhea and EBF are present in the first 6 weeks after birth (53). Thus, in settings like Egypt, a previous study has shown an inverse relationship between EBF and modern contraceptive uptake, suggesting that prolonged breastfeeding may delay the adoption of hormonal or barrier methods (54). Postpartum women should be provided with accurate information about LAM, along with guidance on when and how to transition to other effective contraceptive options beyond the LAM window. Findings of presented study underscore the necessity of integrating FP and breastfeeding counseling into a unified, proactive, and continuous service model. This integration should be systematically implemented within a single center, ensuring that postpartum women receive consistent and periodic guidance on both LAM and subsequent contraceptive options. Such an approach would enhance the continuity of care, address the evolving contraceptive needs of mothers, and support informed decision-making throughout the postpartum period.

### 4.6 Seasonal work

In the Şanlıurfa region, our data highlight the impact of economic hardship and inequalities in formal employment on inadequate breastfeeding outcomes. The findings of this study revealed a significant association between maternal involvement in seasonal agricultural work and shorter durations of EBF. A recent systematic review by Gupta also clearly reported the negative effects of agricultural work such on nutrition outcomes (55).

In line with literature (56) our findings also revealed that women doing seasonal agricultural work are inadequate in terms of contraception and use of modern methods, and this situation negatively affects ideal breastfeeding. Women were reported to return to physically demanding work shortly after childbirth, with limited workplace flexibility, no maternity leave, and poor lactation accommodations It is inevitable that seasonal agricultural work will negatively affect effective contraception and ideal breastfeeding under all adverse conditions such as exposure to physical and mental factors (57) In this period when access to health institutions will be difficult, providing breastfeeding support and FPCs with structured mobile health services may be a solution.

### 4.7 Studies on interventions

A recent Systematic Review and Meta-Analysis (2025) of 34 randomized and experimental studies (n = 4,698) to assess antenatal interventions aimed at improving breastfeeding self-efficacy by Koruk et al. found that model-based education combined with counseling yielded the greatest effect size, outperforming standard education alone and simulation or counseling without prior education (58).

Wong et al. suggest multicomponent, theory-based intervention with ≥3 sessions delivered via both face-to-face teaching and telephone follow-ups across antenatal and postnatal period can be effective to enhance EBF over 6-month, partial breastfeeding and breastfeeding self-efficacy over 2-month postpartum (59). Breastfeeding counseling given by a lactation consultant for up to 10 weeks has been shown to provide significantly successful results for EBF, breastfeeding maintenance, and effective breastfeeding compared to the control group (21). Support given via WhatsApp messaging application is shown to significant positive effect on breastfeeding durations (60). Chipojola et al. (2022) show that breastfeeding promotion programs are effective in increasing EBF rates in the early postpartum period (<1 month), but that for breastfeeding to be sustained through months 1–5, multifaceted interventions with continuity are required (61). Pezley at al. and Gavine at al. supported the field by offered interventions (62, 20).

Studies have shown that structured breastfeeding support services have significant positive contributions to breastfeeding rates (63–67). However, if improvements cannot be made in women's reproductive problems and the accompanying domestic, physical and psychological problems, reaching the desired target in breastfeeding rates does not seem easy at all. The most reasonable solution seems to be to plan interventions suitable for the regional structure and implement them decisively.

### 4.8 Limitations

While this study offers important insights into breastfeeding barriers in Şanlıurfa, several limitations should be acknowledged:

1. Retrospective Design: The study relies on medical and institutional records collected in the past. Such data sources may have missed, incomplete, or inconsistently recorded elements.
2. Single Location/Single Center: All data were gathered from one support center in Şanlıurfa. Therefore, findings may not be generalizable to the broader population of Türkiye, rural vs. urban areas, or to other regions with different cultural, healthcare access, or socioeconomic conditions.
3. Variable Limitations: Not every potentially relevant factor was measured. Variables such as family/community social support, economic strain, maternal psychological state (e.g., depression), detailed obstetric history beyond mode of birth (e.g., complications), or workplace constraints may be under-measured or missing entirely.
4. Representativeness Concerns: The sample has very high rates of cesarean births, miscarriages, low maternal education, and low contraceptive use. Such extreme values may reflect circumstances of the study center and may not represent the “average” mother in Şanlıurfa or wider Türkiye.
5. Possible Measurement Bias: Key outcomes may depend on self-reporting, or maternal recall, which can be subject to recall bias.

### 4.9 Implications and recommendations

Based on these findings, the following implications and recommendations are proposed for policy makers and health program planners in Şanlıurfa to reduce breastfeeding barriers and improve EBF rates:

1. Enhance Prenatal Education and Health Literacy

It is recommended that targeted maternal education programs be implemented during antenatal visits, especially for women with low formal education. The education content should include the benefits of EBF, strategies to manage breastfeeding following cesarean delivery, and information on contraception. Visual aids, and instruction in local languages or dialects, may improve comprehension. Health education interventions might also begin during late adolescence and ideally be incorporated formally into school curricula.

1. Enhance BSCs

It is recommended that BSC staffed by certified and trained professionals be expanded, with a focus on providing dedicated lactation counselling and follow-up care. These centres should specialize in breastfeeding management (including after cesarean delivery), provide ongoing emotional and technical support to mothers, and be accessible through primary healthcare facilities. To ensure effectiveness and sustainability, these centers should employ fully certified lactation consultants, integrate services with postnatal and community health follow-up, and ensure continuity of care beyond hospital discharge.

1. Strengthen FP and Contraceptive Access

It is recommended that breastfeeding counselling be integrated with FPCs, so mothers understand how correct contraceptive use helps birth spacing, which in turn supports sustained EBF. To avoid any neglect in implementing these steps due to time constraints or lack of motivation, service planning should include regular monitoring, accountability mechanisms, and dedicated staff-time for joint counselling.

1. Address Socioeconomic and Occupational Barriers

It is recommended that interventions be developed to accommodate the realities of seasonal work among agricultural or labourer populations—such as offering flexible schedules and providing facilities for breastfeeding or milk expression at workplaces. Additionally, mobile service delivery should be considered, bringing breastfeeding counselling and FPCs directly to seasonal workers to reduce access barriers due to time constraints or geographic isolation. Policymakers should promote the deployment of community health workers or peer counsellors to provide ongoing social support for mothers, particularly those who lack family support or must return to work soon after childbirth.

1. Policy and Health System Interventions

It is recommended that “Pregnancy School” programs be reinforced and expanded, ensuring that their curricula explicitly include modules on breastfeeding (especially EBF), different childbirth methods (including cesarean vs. vaginal birth), FP, and comprehensive postnatal support. Such programs should be accessible to all expectant mothers and incorporate partners or family members where possible, to foster supportive environments for maternal and infant health.

1. Monitoring, Research and Local Data

It is recommended that local longitudinal studies or follow-up mechanisms be established in Şanlıurfa to assess how interventions—such as education programs, hospital practices, and FPCs—affect breastfeeding initiation, exclusivity, and duration over time. Policy makers are encouraged to monitor trends in mode of delivery (vaginal vs. cesarean), contraceptive use, and maternal education, linking these data to breastfeeding outcomes to evaluate the effectiveness of policy changes and program implementations.

1. Community Engagement

It is recommended that collaborations be established with local community leaders, elder women, and religious figures to foster an environment where breastfeeding practices are embraced as social norms. Such partnerships can play a pivotal role in reducing the stigma mothers may face when breastfeeding or utilizing contraceptive methods. Engaging these respected community members can facilitate culturally sensitive education and support, thereby enhancing the acceptance and practice of breastfeeding and FP within the community.

1. Implementing Peer Support Networks

The establishment of peer mother support groups in villages and peri-urban areas is encouraged. These groups, led by trained peer counselors, can provide a platform for mothers to share experiences, offer encouragement, and receive guidance, particularly benefiting those with lower educational backgrounds or those engaged in seasonal work.

## 5 Conclusion

In conclusion, this study carried out in Şanlıurfa reveals several interrelated determinants hampering optimal breastfeeding outcomes. High rates of cesarean births were associated with delayed initiation of breastfeeding within the first hour after birth. Contraceptive use among mothers was also very low, despite high rates of traditional methods. The findings suggest that mothers with low education, those not using modern contraception, those who delivered via caesarean section, and seasonal workers face particular risk of early cessation or suboptimal breastfeeding. These results underscore that breast-feeding in this region is not merely a matter of maternal intention but is heavily influenced by obstetric, educational, socioeconomic, and reproductive health factors.

Integrating FPCs into BSCs, especially in regions with high fertility rates, low levels of education and where sociocultural norms are decisive on attitudes, can be a rational approach that can improve maternal and child health by positively impacting breastfeeding outcomes.

## Data Availability

The data that support the findings of this study are available from the author upon reasonable request.

## References

[1] Walters DD Phan LTH Mathisen R. The cost of not breastfeeding: global results from a new tool. Health Policy Plan. (2019) 34:407–17. 10.1093/heapol/czz050 31236559 PMC6735804

[2] Victora CG Bahl R Barros AJD França GVA Horton S Krasevec J Breastfeeding in the 21st century: epidemiology, mechanisms, and lifelong effect. Lancet. (2016) 387(10017):475–90. 10.1016/S0140-6736(15)01024-7 26869575

[3] World Health Organization. Exclusive breastfeeding for optimal growth, development, and health of infants (2023). Available online at: https://www.who.int/tools/elena/interventions/exclusive-breastfeeding (Accessed May 9, 2025).

[4] The United Nations Children's Fund (UNICEF). Global breastfeeding scorecard. Rates of breastfeeding increase around the world through improved protection and support. (2023). Available online at: https://www.unicef.org/documents/global-breastfeeding-scorecard-2023 (Accessed April 05, 2025).

[5] World Health Organization. Infant and young child feeding (2023). Available online at: https://www.who.int/news-room/fact-sheets/detail/infant-and-young-child-feeding (Accessed March 22, 2025).

[6] Springall TL McLachlan H Forster DA Browne J Chamberlain C. Breastfeeding rates of aboriginal and torres strait islander women in Australia: a systematic review and narrative analysis. Women Birth. (2022) 35(6):e624–38. 10.1016/j.wombi.2022.02.011 35288036

[7] O'Connell MA Meedya S Al Baqali J Alraeesi K Leahy-Warren P. A systematic review and meta-analysis of breastfeeding rates, factors influencing breastfeeding and practices in the United Arab Emirates (UAE). Int Breastfeed J. (2025) 20(1):37. 10.1186/s13006-025-00728-2 40380254 PMC12085000

[8] Moret-Tatay A Pérez-Bermejo M Asins-Cubells A Moret-Tatay C Murillo-Llorente MT. A systematic review of multifactorial barriers related to breastfeeding. Healthcare (Basel). (2025) 13(11):1225. 10.3390/healthcare13111225 40508839 PMC12154042

[9] ACOG. Committee opinion: barriers to breastfeeding: supporting initiation and continuation of breastfeeding:, number 821. Obstet Gynecol. (2021) 137(2):e54–62. 10.1097/AOG.0000000000004249 33481532

[10] The United Nations Children's Fund (UNICEF). Breastfeeding is not Just a one-woman job. It requires encouragement and support from skilled counsellors, family members, health care providers, Employers, Policymakers and Others (2019). Available online at: https://www.unicef.org/armenia/en/stories/breastfeeding-not-just-one-woman-job (Accessed December 10, 2024).

[11] World Health Organization. Family Planning/Contraception Methods. (2023). Available online at: https://www.who.int/news-room/fact-sheets/detail/family-planning-contraception#:∼:text=methods%20of%20contraception.-,Contraceptive%20methods,and%20fertility%20awareness%2Dbased%20methods (Accessed February 06, 2024)

[12] Hu D Tang Y Pei K. Strategies for improving postpartum contraception compared with routine maternal care: a systematic review and meta-analysis. Int J Public Health. (2023) 68:1605564. 10.3389/ijph.2023.1605564 37124160 PMC10133502

[13] Centers for Disease Control and Prevention. Contraceptive use during lactation (2023). Available online at: https://stacks.cdc.gov/view/cdc/162370 (Accessed May 9, 2025).

[14] United Nations. Department of Economic and Social Affairs. New York, NY: Population Division World Fertility and Family Planning (2020). Available online at: https://www.un.org/en/development/desa/population/publications/pdf/family/World_Fertility_and_Family_Planning_2020_Highlights.pdf (Accessed December 10, 2024).

[15] Kehinde J O'Donnell C Grealish A. The effectiveness of prenatal breastfeeding education on breastfeeding uptake postpartum: a systematic review. Midwifery. (2023) 118:103579. 10.1016/j.midw.2023.103579 36580847

[16] Oggero MK Rozmus CL LoBiondo-Wood G. Effects of prenatal breastfeeding education on breastfeeding duration beyond 12 weeks: a systematic review. Health Educ Behav. (2024) 51(5):665–76. 10.1177/10901981231220668 38240358 PMC11420594

[17] Blazer C Prata N. Postpartum family planning: current evidence on successful interventions. Open Access J Contracept. (2016) 7:53–67. 10.2147/OAJC.S98817 29386937 PMC5683159

[18] Turkish Demographic and Health Survey (2018). Available online at: https://fs.hacettepe.edu.tr/hips/dosyalar/Arastirmalar%20-%20raporlar/2018%20TNSA/TNSA2018_ana_Rapor_compressed.pdf (Accessed January 01, 2025).

[19] Sinha B Chowdhury R Sankar MJ Martines J Taneja S Mazumder S Interventions to improve breastfeeding outcomes: a systematic review and meta-analysis. Acta Paediatr. (2015) 104:114–34. 10.1111/apa.13127 26183031

[20] Gavine A Shinwell SC Buchanan P Farre A Wade A Lynn F Support for healthy breastfeeding mothers with healthy term babies. Cochrane Database Syst. Rev. (2022) 10:CD001141. 10.1002/14651858 36282618 PMC9595242

[21] van Dellen SA Wisse B Mobach MP Dijkstra A. The effect of a breastfeeding support programme on breastfeeding duration and exclusivity: a quasi-experiment. BMC Public Health. (2019) 19(1):993. 10.1186/s12889-019-7331-y 31340787 PMC6657127

[22] Pratiwi R Atmaka DR Sutoyo DAR Mahmudiono T. The effectiveness of smartphone-based nutrition education intervention in successful practice of exclusively breastfeeding: a meta-analysis. Amerta Nutrition. (2023) 7(4):615. 10.20473/amnt.v7i4.2023.615-625

[23] Turkish Statistical Institute (TUIK). Birth statistics. (2022). Available online at: https://data.tuik.gov.tr/Bulten/Index?p=Dogum-Istatistikleri-2021-45547 (Accessed January 07, 2025).

[24] World Health Organization. Leaving No One Behind: WHO spearheads health services for Syrian refugees in Turkey, Türkiye. Geneva. (2021). Available online at: https://www.who.int/about/ accountability/results/who-results-report-2020-mtr/country-story/2021/turkey (Accessed September 05, 2025).

[25] Koruk İ Gökçeoğlu S Allahverdi Ş Kuzan R. Level of exclusive breastfeeding for the first 6 months in 0–24-month infants and children in a family health center in Şanlıurfa and the factors affecting it. J Health Sci Res. (2019) 4(1):21–31. 10.18311/jhsr/2019/23330

[26] Er B Koçakoğlu Ş. Şanlıurfa İli Merkez İlçelerinde 0–24 aylık bebeği olan annelerin anne sütü ve emzirmeye İlişkin bilgi ve tutumları. Harran Üniv Tıp Fakültesi Dergisi. (2022) 19(3):542–50. 10.35440/hutfd.1205671

[27] Güner Ö Koruk F. Şanlıurfa'da 0–6 aylık bebeklerin sadece anne sütü alma durumları ve etkileyen faktörler. Harran Üniv Tıp Fakültesi Dergisi. (2019) 16(1):111–16.

[28] Ortaç G Koruk F. Şanlıurfa’da postpartum dönemde kadınların aile planlamasına yönelik tutum ve davranışları. Gazi Sağlık Bilimleri Dergisi. (2024) 9(2):54–65. 10.52881/gsbdergi.1421328

[29] Sanliurfa Il Saglik Müdürlügü. Egitim ve Arastirma Hastanesi. Dogum Istatistikleri. (2024). Available online at: https://sanliurfaeah.saglik.gov.tr/TR-950434/dogum-istatistikleri.html (Accessed September 05, 2025).

[30] World Health Organization. Department of Reproductive Health and Research (WHO/RHR) and Johns Hopkins Bloomberg School of Public Health/Center for Communication Programs (CCP), Knowledge for Health Project. Family Planning: A Global Handbook for Providers (2018 update). Baltimore and Geneva: CCP and WHO (2018). Available online at: https://cdn.who.int/media/docs/default-source/reproductive-health/contraception-family-planning/mechanisms-of-action-and-effectiveness-of-contraception-methods.pdf?sfvrsn=e39a69c2_1 (Accessed March 02, 2024).

[31] Taha Z Ali Hassan A Wikkeling-Scott L Papandreou D. Factors associated with delayed initiation and cessation of breastfeeding among working mothers in Abu Dhabi, the United Arab Emirates. Int J Womens Health. (2021) 13:539–48. 10.2147/IJWH.S303041 34104003 PMC8180278

[32] Al Sabbah H Assaf EA Taha Z Qasrawi R Radwan H. Determinants of exclusive breastfeeding and mixed feeding among mothers of infants in Dubai and Sharjah, United Arab Emirates. Front Nutr. (2022) 9:872217. 10.3389/fnut.2022.872217 35619950 PMC9127614

[33] Sylvain MH Valens R. Factors associated with postpartum family planning use in Rwanda. Contracept Reprod Med. (2024) 9(1):1. 10.1186/s40834-023-00261-9 38167559 PMC10759325

[34] Ahinkorah BO Budu E Aboagye RG Agbaglo E Arthur-Holmes F Adu C Factors associated with modern contraceptive use among women with no fertility intention in sub-saharan Africa: evidence from cross-sectional surveys of 29 countries. Contracept Reprod Med. (2021) 6(1):22. 10.1186/s40834-021-00165-6 34332644 PMC8325798

[35] Whipps MD. Education attainment and parity explain the relationship between maternal age and breastfeeding duration in U. S. mothers. J Hum Lact. (2017) 33(1):220–4. 10.1177/0890334416679385 28135484

[36] Tang K Wang H Tan SH Xin T Qu X Tang T Association between maternal education and breast-feeding practices in China: a population-based cross-sectional study. BMJ Open. (2019) 9(8):e028485. 10.1136/bmjopen-2018-028485 31467048 PMC6720234

[37] Zhao J Zhao Y Du M Binns CW Lee AH. Maternal education and breastfeeding practices in China: a systematic review and meta-analysis. Midwifery. (2017) 50:62–71. 10.1016/j.midw.2017.03.011 28390256

[38] Dagnew GW Asresie MB Fekadu GA Gelaw YM. Modern contraceptive use and factors associated with use among postpartum women in Ethiopia; further analysis of the 2016 Ethiopia demographic and health survey data. BMC Public Health. (2020) 20(1):661. 10.1186/s12889-020-08802-6 32398123 PMC7216498

[39] Perrella SL Abelha SG Vlaskovsky P McEachran JL Prosser SA Geddes DT. Australian women’s experiences of establishing breastfeeding after caesarean birth. Int J Environ Res Public Health. (2024) 21(3):296. 10.3390/ijerph21030296 38541296 PMC10969918

[40] Prior E Santhakumaran S Gale C Philipps LH Modi N Hyde MJ. Breastfeeding after cesarean delivery: a systematic review and meta-analysis of world literature. Am J Clin Nutr. (2012) 95(5):1113–35. 10.3945/ajcn.111.030254 22456657

[41] Rowe-Murray HJ Fisher JR. Baby friendly hospital practices: cesarean section is a persistent barrier to early initiation of breastfeeding. Birth. (2002) 29(2):124–31. 10.1046/j.1523-536X.2002.00172.x 12000413

[42] Nakiwunga N Kakaire O Ndikuno CK Nakalega R Mukiza N Atuhairwe S. Contraceptive uptake and associated factors among women in the immediate postpartum period at Kawempe hospital. BMC Womens Health. (2022) 22(1):281. 10.1186/s12905-022-01856-1 35799181 PMC9261026

[43] Zimmerman LA Yi Y Yihdego M Abrha S Shiferaw S Seme A Effect of integrating maternal health services and family planning services on postpartum family planning behavior in Ethiopia: results from a longitudinal survey. BMC Public Health. (2019) 19:1448. 10.1186/s12889-019-7703-3 31684905 PMC6829796

[44] Schafer EJ Campo S Colaizy TT Mulder PJ Breheny P Ashida S. First-time mothers’ breast-feeding maintenance: role of experiences and changes in maternal perceptions. Public Health Nutr. (2017) 20(17):3099–108. 10.1017/S136898001700221X 28879823 PMC10261612

[45] Choedon P Chozom P Rai P Shaiak R Lepcha S Xavier S Assessing knowledge and attitude regarding exclusive breast feeding among primipara mothers. RGUHS J Nurs Sci. (2020) 10(2):88–92. 10.26715/rjns.10_2_6

[46] Agula C Henry EG Asuming PO Obeng-Dwamena A Toprah T Agyekum MW Postpartum contraceptive initiation and use: Evidence from Accra, Ghana. Women’s Health. (2022) 18:1–11. 10.1177/17455057221141290 PMC9742708 36476194

[47] Tesfa D Tiruneh SA Gebremariam AD Azanaw MM Engidaw MT Kefale B The pooled estimate of the total fertility rate in sub-Saharan Africa using recent (2010–2018) demographic and health survey data. Front Public Health. (2023) 10:1053302. 10.3389/fpubh.2022.1053302 36777768 PMC9909402

[48] Labbok MH Hight-Laukaran V Peterson AE Fletcher V Dewey K Lusk D Multicenter study of the lactational amenorrhea method (LAM): I. Efficacy, duration, and implications for clinical application. Contraception. (1997) 55(6):327–36. 10.1016/S0010-7824(97)00040-1 9262927

[49] Uvnäs MK Ekström-Bergström A Buckley S Massarotti C Pajalic Z Luegmair K Maternal plasma levels of oxytocin during breastfeeding-A systematic review. PLoS One. (2020) 15(8):e0235806. 10.1371/journal.pone.0235806 32756565 PMC7406087

[50] Bharathi P. Rachana KM Kaushalya MK Pradeep MRA. Study on knowledge, attitude, awareness and practice of exclusive breast feeding among postnatal mother at a tertiary care hospital. Education. (2024) 35(1):0–95. 10.69605/ijlbpr_13.10.2024.117

[51] Van der Wijden C Manion C, Cochrane fertility regulation group. Lactational amenorrhoea method for family planning. Cochrane Database Syst Rev. (1996) 2015(10):CD001329. 10.1002/14651858.CD001329.pub2 PMC6823189 26457821

[52] Peterson AE Pérez-Escamilla R Labbok MH Hight-Laukaran V Hurtado E Dewey KG Multicenter study of the lactational amenorrhea method (LAM) III: effectiveness, duration, and satisfaction with reduced client-provider contact. Contraception. (2000) 62(4):221–30. 10.1016/S0010-7824(00)00171-2 11172792

[53] Eticha TG Asfaw Y Getachew T Belay Y Alemayehu M Kebede D. Effectiveness of lactational amenorrhea method in Ethiopia: implications for family planning counseling. Open Access J Contracept. (2023) 14:149–57. 10.2147/OAJC.S414436 37693930 PMC10488729

[54] Afifi M. Lactational amenorrhoea and modern contraceptives use among nursing women in Egypt 2003. Oman Med J. (2008) 23(2):72–7. PMID: 22379541. 22379541 PMC3282425

[55] Gupta P. A systematic review on women’s participation in agricultural work and nutritional outcomes. arXiv. 2504.03202. (2025). doi: 10.48550/arXiv.2504.03202

[56] Bucak F Kahraman S. Mevsimlik tarım İşçisi gebe kadınların aile planlamasına yönelik tutumların belirlenmesi. Gevher Nesibe J Med Health Sci. (2020) 5(7):61–6. 10.46648/gnj.89

[57] Cancino J Soto K Tapia J Muñoz-Quezada MT Lucero B Contreras C Occupational exposure to pesticides and symptoms of depression in agricultural workers. A systematic review. Environ Res. (2023) 231:116190. 10.1016/j.envres.2023.116190 37217130

[58] Koruk F Kahraman S Turan Z Nur Özgen H Beyazgül B. The effects of interventions during pregnancy to improve breastfeeding self-efficacy: systematic review and meta-analysis. J Midwif Womens Health. (2025) 70(4):610–23. 10.1111/jmwh.13742 PMC12365724 40128941

[59] Wong MS Mou H Chien WT. Effectiveness of educational and supportive intervention for primiparous women on breastfeeding related outcomes and breastfeeding self-efficacy: a systematic review and meta-analysis. Int J Nurs Stud. (2021) 117:103874. 10.1016/j.ijnurstu.2021.103874 33548592

[60] Sevda KÖ Sevil İ. Continuous lactation support provided through the whatsapp messaging application: a randomized controlled trial. J Hum Lact. (2023) 39(4):666–78. 10.1177/08903344231192948 37646262

[61] Chipojola R Khwepeya M Gondwe KW Rias YA Huda MH. The influence of breastfeeding promotion programs on exclusive breastfeeding rates in sub-Saharan Africa: a systematic review and meta-analysis. J Hum Lact. (2022) 38(3):466–76. 10.1177/08903344221097689 35684942

[62] Pezley L Cares K Duffecy J Koenig MD Maki P Odoms-Young A Efficacy of behavioral interventions to improve maternal mental health and breastfeeding outcomes: a systematic review. Int Breastfeed J. (2022) 17(1):67. 10.1186/s13006-022-00501-9 36064573 PMC9446548

[63] Koçakoğlu Ş Çadirci D. Evaluation of breastfeeding support center applications. Ankara Med J. (2020) 20(1):105–15. 10.5505/amj.2020.97759

[64] Law KH Dimmock JA Guelfi KJ Nguyen T Bennett E Gibson L A peer support intervention for first-time mothers: feasibility and preliminary efficacy of the mummy buddy program. Women Birth. (2021) 34(6):593–605. 10.1016/j.wombi.2020.10.009 33160896

[65] Tseng JF Chen SR Au HK Chipojola R Lee GT Lee PH Effectiveness of an integrated breastfeeding education program to improve self-efficacy and exclusive breastfeeding rate: a single-blind, randomised controlled study. Int J Nurs Stud. (2020) 111:103770. 10.1016/j.ijnurstu.2020.103770–38 32961461 10.1016/j.ijnurstu.2020.103770

[66] Handayani L Prihadi KD Vergawita T Fitriani I Hafidz B Aisyahrani AIB. The role of social-psychological support in breastfeeding promotion among young mothers: literature review. Int J Public Health. (2025) 14(1):148–60. 10.11591/ijphs.v14i1.25515

[67] Gamit R Jomy R Rathod H Abrahams N Patel B Mathew M A study to assess the effectiveness of structured teaching programme on knowledge regarding breast feeding among postnatal mothers in Shri Vinoba Bhave, Civil Hospital, Silvassa. Int J Adv Nurs Manag. (2021) 9(1):4–6. 10.5958/2454-2652.2021.00002.0

