# Supplementary material for: The link between breastfeeding profile and contraceptive use and barriers to breastfeeding: messages from a breastfeeding support center on the need for further strategies to improve breastfeeding
Source: Front Pediatr. 2025 Nov 6;13:1633498. doi: 10.3389/fped.2025.1633498 (PMC12631282; doi:10.3389/fped.2025.1633498)
Supplement: Supplementary file 1 [file Datasheet1.pdf]

	T.C.<br>ŞANLIURFA HARRAN ÜNİVERSİTESİ HASTANESİ<br>EMZİRME DESTEK MERKEZİ HASTA BİLGİ FORMU			
Kodu	Yayın Tarihi	Revizyon No	Revizyon Tarihi	Sayfa No
YD.FR.33	29.08.2018	22.11.2022	---	1 / 2

Anne Adı:………………………………….............. **Anne Yaşı:**…. **Meslek:** ……………….............................. **T.C. :**……………………............

Annenin Eğitim Durumu: Okuryazar Değil ☐ İlkokul ☐ Ortaokul ☐ Lise ☐ Üniversite ☐ Yüksek Lisans ☐

Anadili: ……………………….. Türkçe Konuşabiliyor ☐ Tercüman Gerekiyor ☐

Eşin Adı :……………..………… **Eşin Mesleği:** ……………..……..

Eşin Eğitim Durumu: Okuryazar Değil ☐ İlkokul☐ Ortaokul ☐ Lise ☐ Üniversite ☐ Yüksek Lisans ☐

İletişim (adres/tel):

Mevsimlik işçiliğe gidecek mi? Evet ☐ Hayır ☐

Anne Adet Gördü mü? : Evet ☐ Hayır ☐ **Adet Görme Zamanı:** ……………….

Korunma Yöntemi: Geri Çekme ☐ Kondom ☐ Hap ☐ RİA ☐ Emzirme ☐ Geleneksel Yöntemler ☐ Korunmuyor ☐ Eşi İstiyor ☐ Kendi İstiyor ☐ Korunmayı Bilmiyorum ☐

Kaçıncı Gebelik:…………… **Kaç Düşük:**……......…… **Normal Doğum Sayısı** ………… **Sezaryen Sayısı** …………..

Doğum Sonrası İlk Emzirme Zamanı: 30 dakika ☐ 1.saat ☐ > 1saat ☐

Kardeşlerin Tek Başına Anne Sütü Süresi: Yok ☐ <30 gün☐ <60 gün ☐ 2-4 ay☐ 4-6 ay☐

Evde emzirmeye destek olan biri var mı? Evet ☐ Hayır ☐

BAŞVURU NEDENİ:

Süt Azalması ☐ Süt Kesilmesi ☐ Bebeğin Kilo Alamaması ☐ Doğum Sonrası Bebeğin Hastanede Yanlış Mama Başlanması ☐

Meme Reddi ☐ Meme Sorunu **[**Meme Ucu Yara ☐ Dolu Meme ☐ Apse ☐ Mastit ☐**]** Memeyi kavrayamama ☐

Bebekte Kilo Kaybı ☐ Bebekte Zayıf Emme ☐ Bebeğin Memede Uyuması ☐ Silikon Uç ☐ Fazla Süt ☐ Evlat Edinme ☐ Diğer ☐

GÖNDEREN:

Bebek Odası ☐ Yenidoğan ☐ Yenidoğan Plk ☐ Sağlam Çocuk ☐ Genel Plk ☐ Acil ☐

Gastro☐ İnternet☐ Arkadaş ☐ Dış Merkez ☐ Diğer ☐

EMZİRME ÖYKÜSÜ:

Doğumdan Sonraki İlk Bir Hafta Süt Miktarı: <10 ml ☐ >10 ml ☐ Bilinmiyor☐

Şimdiki Süt Miktarı: <10 ml ☐ 10-30ml ☐ 30-50 ml ☐ ≥ 50 ml ☐ Bilinmiyor ☐

Emzirme Sorunu: Var ☐ Yok☐

Bebeğin Adı Soyadı:……………………………………………………………………………………………

Cinsiyeti: **Kız** ☐ **Erkek** ☐ **Tekiz** ☐ **İkiz** ☐

Doğum Tarihi: …………………………………… **Takvim Yaşı :** ………………………………

Gebelik Haftası: Term (>37) ☐ **Geç preterm(35-37)** ☐ **Preterm<35** ☐ **Düzeltilmiş Yaşı:** ……………………………

Doğum Yeri: Üniversite Hastanesi ☐ Devlet Hastanesi ☐ Özel ☐

Doğum Şekli: C/S ☐ NSVY ☐ **Doğum Ağırlığı:**…………………………… **SGA** ☐ **LGA** ☐ **AGA** ☐

Kadın Doğum Taburculuk Tarihi: ………………………………………… **Kadın Doğum Taburculuk Kilosu:** ………

Doğum Ağırlığını Yakalama Zamanı: ≤14 gün ☐ >15 gün ☐

Hastanede Yatış: Evet ☐ Hayır ☐ **SÜRE:** <15GÜN ☐ >15GÜN ☐ >31 GÜN ☐

Tanı: Prematürite ☐ SGA ☐ Enfeksiyon ☐ Sepsis ☐ Sarılık ☐ Kilo Kaybı ☐ Dehidratasyon ☐ HİE ☐ Anomali ☐

Yenidoğan Taburculuk Tarihi: ……………………………………………**Yenidoğan Taburculuk Kilosu:**..

Mamaya İlk Başlama Zamanı: <7GÜN ☐ 7-14 GÜN☐ 1.AY ☐ 2.AY ☐ 3.AY ☐ >4.AY ☐

Mamaya Başlayan: Üniversite ☐ Doğumevi ☐ Özel Hastane ☐ Muayenehane ☐ Aile Hekimi ☐ Hemşire ☐

Aile Yakını ☐ Kendisi ☐

Mama Başlama Nedeni:

C/S Nedeniyle Süt Yok ☐ Kilo Alımı Az ☐ Kilo Kaybı Çok ☐ Sarılık ☐ Doymuyor Endişesi ☐ Diğer☐

Mama Sayı: <8☐ ≥8☐

Öğün Başına Mama Miktarı: <30ML☐ 30-50 ml☐ > 50 ml ☐

Emmeyi Tam Başarıyor mu? : Evet ☐ Hayır ☐

Ne Zamandır Emmeyi Tam Başaramıyor: Doğumdan beri ☐ < 1ay ☐ 1-2 ay ☐ 2-3 ay ☐ > 3 ay ☐

Annenin Hastalığı/Kullandığı İlaç:

Bebeğin Hastalığı /Kullandığı İlaç:

NOTLAR:
